# Utility of SUVmax on Primary Staging 68Ga‐PSMA PET/CT in Determining Metastatic Status of Prostate Cancer

**DOI:** 10.1111/1754-9485.70081

**Published:** 2026-03-09

**Authors:** Geoffrey Liu, Shane Lee, Kabir Ahmad

**Affiliations:** ^1^ Barwon Medical Imaging, Barwon Health Geelong Australia; ^2^ Research Development Unit, Barwon Health Geelong Australia

**Keywords:** metastasis, prostate cancer, PSMA PET, staging, SUVmax

## Abstract

**Introduction:**

68Ga prostate‐specific membrane antigen (PSMA) has been shown to have prognostic value in detecting PSMA expressing metastasis from prostate cancer, but inevitably, there are cases in which suspected metastases demonstrate equivocal uptake. This study aims to assess whether there is a direct relationship between SUVmax of the primary prostate lesion and the presence of nodal and distant metastasis.

**Methods:**

Retrospective review of 265 primary staging 68Ga‐PSMA PET/CT studies performed at a single centre on a single PET/CT was performed for the SUVmax of the primary lesion, and the presence of PSMA‐avid nodal/distant metastases.

**Results:**

Analysis shows that a single unit increase in SUVmax of the primary prostatic lesion corresponds with a 1.6% increased odds of nodal metastases, 1.7% increased odds of distant metastases and a 2% increased odds of having either. A single unit increase in the log of the SUVmax corresponds with odds ratios of 2.061, 2.038 and 2.294 respectively.

**Conclusion:**

There is a weak but statistically significant correlation between the SUVmax of the primary prostate lesion and the presence of metastases on primary staging 68Ga‐PSMA PET/CT for biopsy‐proven prostate cancer. However, because the relationship is so weak and the significant overlap in SUVmax in local versus advanced disease, using SUVmax as the sole predictor of advanced disease is unreliable.

## 1 Introduction

Prostate cancer is the second most common cancer to affect the male population worldwide and the fifth most common cause of cancer deaths worldwide [1]. This results in a large morbidity and mortality burden on the population and significant cost to the health system in managing this disease. Accurate and prompt staging of prostate cancer is vital for the appropriate management of patients.

PSMA PET is a relatively new development in the imaging of prostate cancer targeting a glutamate carboxypeptidase type II non‐secreted transmembrane protein, which is expressed in over 95% of prostate cancers [2]. This results in a nucleotide with an 85.9% sensitivity and 99.5% specificity in detecting metastatic nodes [3].

PSMA PET was initially introduced into our department in 2018 and has been used for both primary staging and restaging of patients with biopsy‐proven prostate cancer. Despite the high sensitivity of 68Ga‐PSMA PET/CT, there are inevitable cases where mild activity in locoregional nodes or at distant sites is equivocal for metastatic disease. This could significantly alter patient management and outcomes. The aim of this retrospective cohort study was to correlate any relationship between the SUVmax of the primary lesion on primary staging 68Ga‐PSMA PET/CT and status of nodal and distant metastasis, hopefully allowing better diagnostic certainty in interpreting equivocal disease. Henceforth, all references to SUVmax refer to the primary prostate lesion on primary staging 68Ga‐PSMA PET/CT.

## 2 Material and Methods

### 2.1 Inclusion Criteria

Between August 2018 (when PSMA PET was first introduced to the department) and December 2023, 298 primary‐staging 68Ga‐PSMA PET/CTs were performed at the Nuclear Medicine Department of Barwon Medical Imaging, University Hospital Geelong, Australia. The inclusion criteria were treatment‐naive, biopsy‐proven patients with prostate cancer (Gleason score greater than 6) who were referred for primary‐staging 68Ga‐PSMA PET/CT. Exclusion criteria included patients who had a synchronous malignancy, those in whom a PSMA‐avid primary lesion was not identified, and those with equivocal nodal or distant metastasis on imaging. Patients with equivocal metastases were excluded as the results could be artefactual or represent micrometastatic disease and may confound any findings.

Of the 298 patients, 33 were excluded due to the exclusion criteria, resulting in 265 patients who underwent data analysis. Of the remaining patients, 71 were demonstrated to have PSMA‐avid nodal metastasis and 55 had PSMA‐avid distant metastasis.

### 2.2 68Ga‐PSMA PET/CT

All 68Ga‐PSMA PET/CT scans were performed following 4 h of fasting and approximately 60 min following intravenous administration of 2 MBq per kilogram of body weight with a dose range of 100–250 MBq. All patients were scanned on a Siemens Biograph mCT‐S(64) with Flow, and diagnostic CT with contrast was performed at the same time for structural analysis. The patients were scanned from the skull vertex to the mid thigh for PET/CT and diagnostic contrast‐enhanced CT of the chest, abdomen and pelvis. The PET data were reconstructed using TrueX + TOF. The diagnostic CT were interpreted with a 4 mm slice thickness.

### 2.3 Image Analysis

All PET/CT scans were interpreted by a dual‐qualified radiologist and nuclear medicine imaging specialist at the time of scanning with repeat SUVmax measurements obtained at the time of data collection. Imaging analysis was performed on Syngo.via (Siemens Healthcare) in both instances. Primary lesions were assessed for the SUVmax with comparison to the background prostate without a predefined SUVmax cut‐off. Lymph nodes and distant metastasis were assessed on both structural appearances and PSMA uptake, then described accordingly. Any patients without a definite PSMA‐avid primary disease were excluded.

### 2.4 Data Analysis

Data points that were collected for each patient include the SUVmax of the primary prostate cancer, as well as the presence or absence of nodal and distant metastases. The data underwent descriptive analysis and were interpreted with a box plot, including median, maximum, minimum, and 1st and 3rd quartiles. Further univariate linear regression and logistic regression analysis were also performed. Data analysis was performed by a qualified biostatistician at the institution where the study was performed. *p* values of less than 0.05 were considered statistically significant. The data was not adjusted for other factors, such as PSA level, Gleason score and ISUP grade group to minimise confounding.

## 3 Results

### 3.1 SUVmax

**FIGURE 1:**
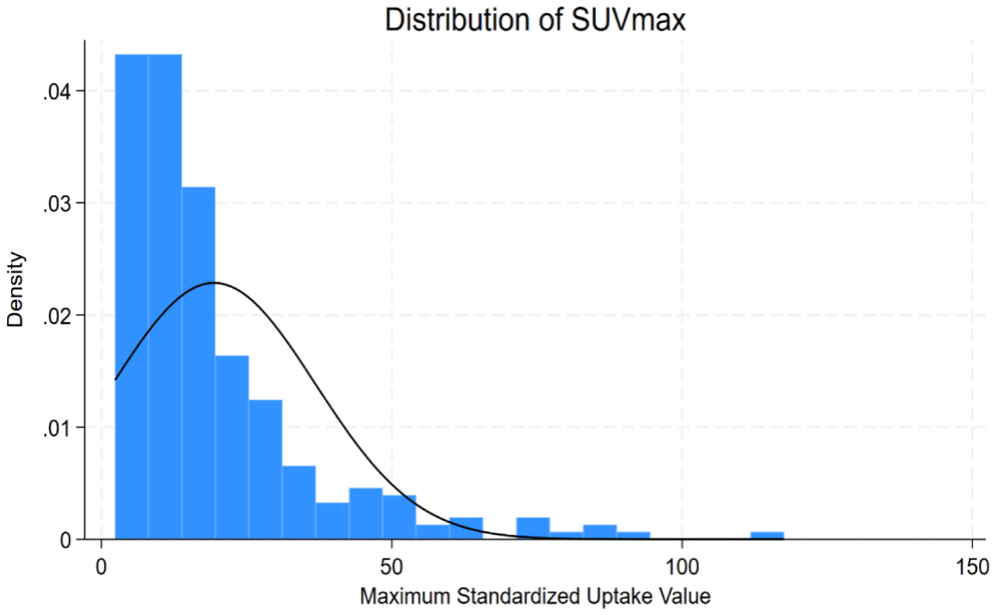
Distribution of SUVmax on primary staging 68Ga‐PSMA PET/CT of the primary prostate cancer.

**FIGURE 2:**
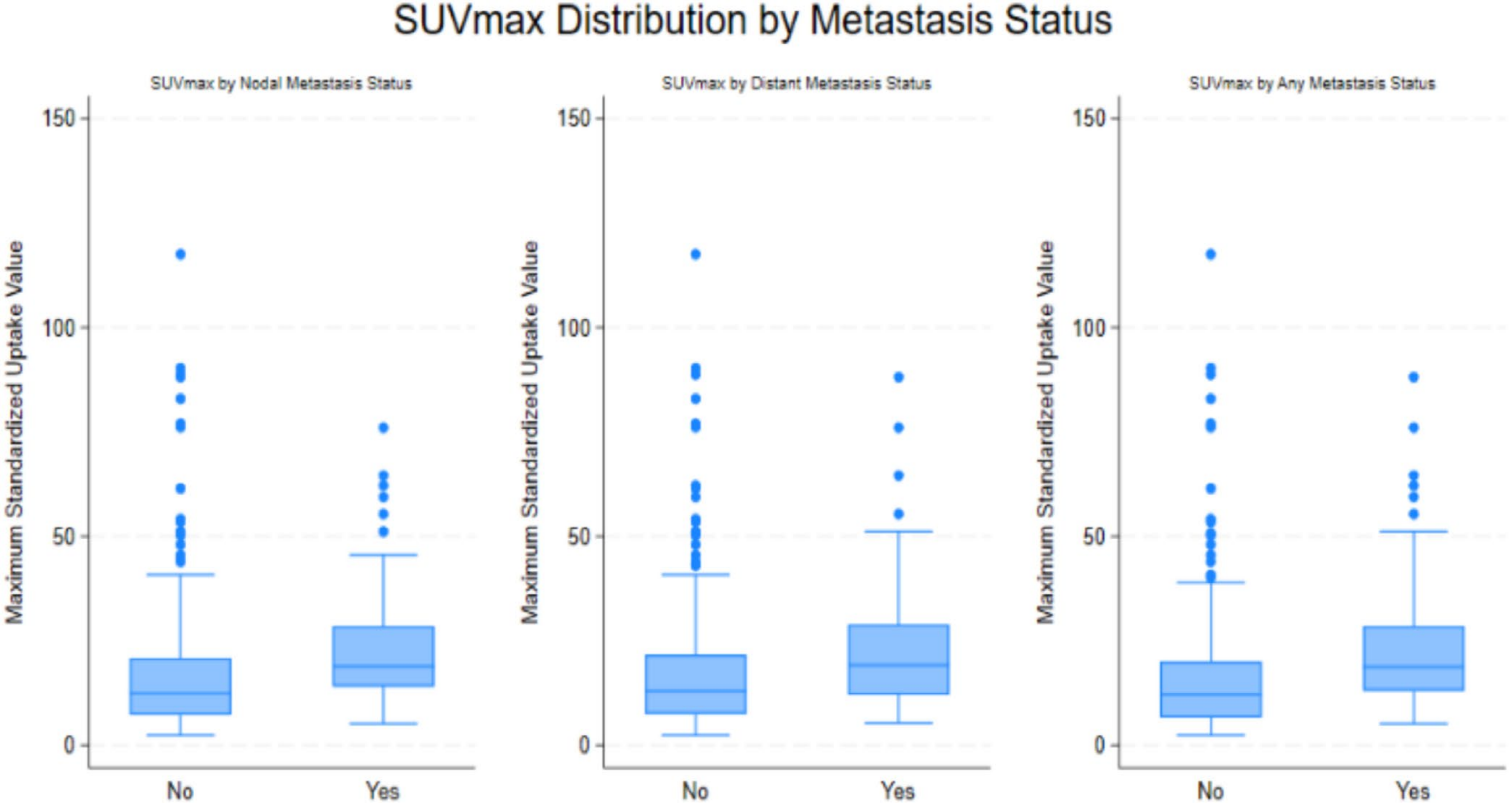
Spread of SUVmax of the primary prostate cancer following stratification by metastatic status.

All 265 patients who were analysed in the study demonstrated focal or diffuse uptake within the prostate gland noting that 11 patients were initally excluded due to the absence of a PSMA‐avid primary despite biopsy‐proven disease. A wide range of SUVmax was demonstrated in the primary prostatic disease ranging from 2.3 to 117 with the greatest proportion of patients demonstrating a lower SUVmax (Figures 1 and 2).

### 3.2 Nodal Metastasis

Of the 265 patients who underwent data analysis, 65 (approximately 26% of the patient cohort) were demonstrated to have PSMA‐avid nodal metastatic disease on primary staging 68Ga‐PSMA PET/CT, the majority involving the iliac/pelvic nodes. The median SUVmax of the primary prostate cancer of those patients without metastatic disease was 12.3 compared with 18.8 for patients with PSMA‐avid nodal metastases. The mean SUVmax was 18.0 versus 23.4, respectively. Both of these findings were clinically significant with a *p*‐value of < 0.001 (Table 1).

**TABLE 1:** Distribution of SUVmax based on nodal and distant metastatic status.

Variables	SUVmax values									
	Mean	Median	SD	Min	Max	*p*25	*p*75	IQR	*N*	*p*
Nodal metastasis										
No	18.0	12.3	18.0	2.3	117.6	7.2	20.8	13.6	196	< 0.001
Yes	23.4	18.8	15.2	5.1	76.0	14.0	28.5	14.5	69	
Distant metastasis										
No	18.1	12.9	17.4	2.3	117.6	7.4	21.6	14.2	210	< 0.001
Yes	24.2	19.1	17.1	5.3	88.1	12.0	28.9	16.9	55	
Nodal or distant metastasis										
No	17.3	12.0	17.6	2.3	117.6	6.5	20.0	13.5	180	< 0.001
Yes	23.8	18.7	16.3	5.1	88.1	13.0	28.5	15.5	85	
Total	19.4	13.9	17.5	2.3	117.6	8.3	24.0	15.7	265	

**FIGURE 3:**
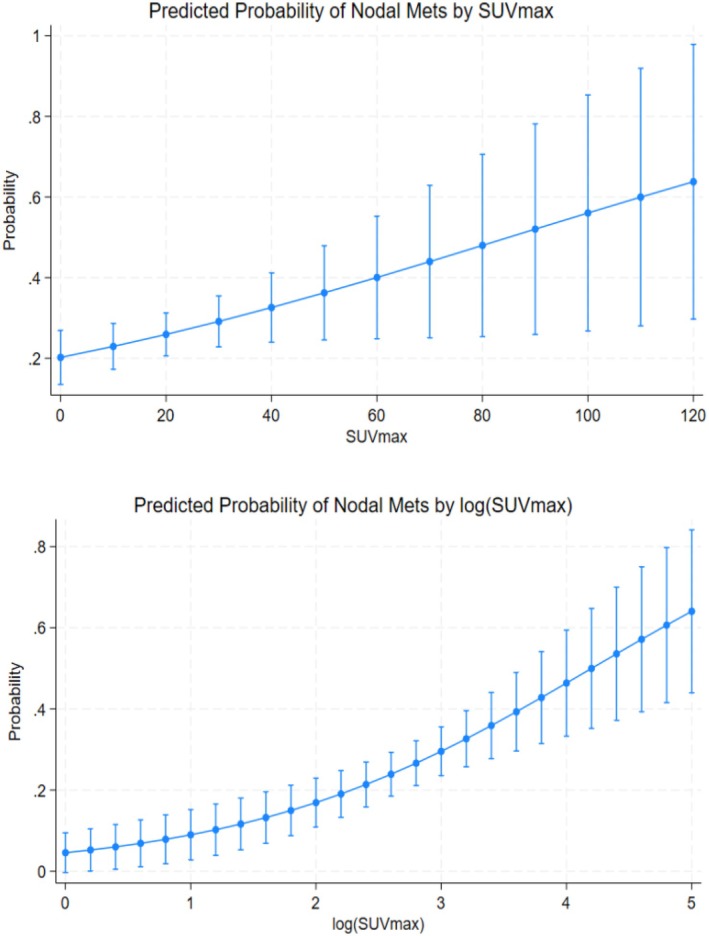
Linear and logarithmic analysis of SUVmax correlated with the probability of nodal metastasis (with 95% confidence intervals).

Further linear and logarithmic regression models were performed with the data which demonstrated that a 1‐unit increase in SUVmax corresponds with a 1.6% increase in odds of having nodal metastasis (*p*‐value 0.031). The log‐transformed SUVmax value had an even stronger correlation with a 1‐unit increase in the log of SUVmax corresponding with a doubling (odds ratio 2.061) of the odds of having nodal metastases (*p*‐value < 0.001) (Table 2 and Figure 3).

**TABLE 2:** Linear and logarithmic regression analysis of data correlating SUVmax and metastatic status.

Model	Outcome variable	*n*	Variable	Coeff	OR (95% CI)	*p*
Model 1	Nodal metastasis (yes)	265	SUVmax	0.016	1.016 (1.001–1.031)	0.031
Model 2	Distant metastasis (yes)	265	SUVmax	0.017	1.017 (1.002–1.033)	0.027
Model 3	Nodal or distant metastasis (yes)	265	SUVmax	0.020	1.020 (1.006–1.036)	0.007
Model 4	Nodal metastasis (yes)	265	Log of SUVmax	0.723	2.061 (1.406–3.019)	< 0.001
Model 5	Distant metastasis (yes)	265	Log of SUVmax	0.712	2.038 (1.356–3.061)	0.001
Model 6	Nodal or distant metastasis (yes)	265	Log of SUVmax	0.830	2.294 (1.580–3.330)	< 0.001

It was noted, however, that nodal metastasis was noted in patients with an SUVmax of as low as 5.1 while a patient with an SUVmax of 117 did not demonstrate nodal metastases (Table 1 and Figure 2).

### 3.3 Distant Metastasis

Of the included 265 patients, 55 (approximately 21% of the patient cohort) were demonstrated to have PSMA‐avid distant metastases, predominately osseous metastases. The median SUVmax of the primary prostate cancer of patients without metastatic disease was 12.9 compared with 19.1 for patients with PSMA‐avid nodal metastases. The mean SUVmax was 18.1 versus 24.2, respectively. Both of these findings were clinically significant with a *p*‐value of < 0.001 (Table 1).

**FIGURE 4:**
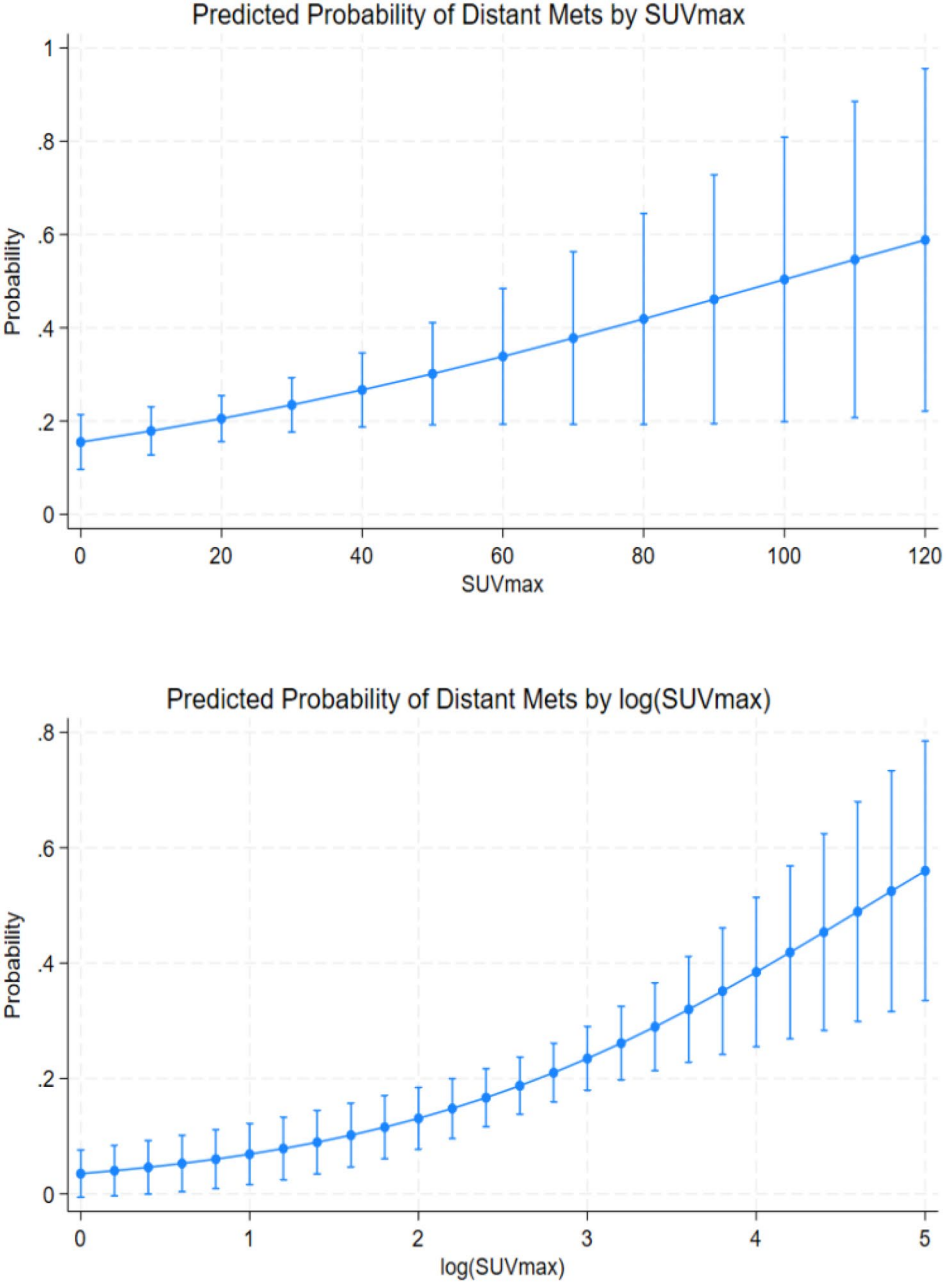
Linear and logarithmic analysis of SUVmax correlated with the probability of distant metastasis (with 95% confidence intervals).

Furthermore, linear regression analysis of the data demonstrated that a 1‐unit increase in the SUVmax of the primary lesion was associated with a 1.7% increase in the odds of having distant metastatic disease (*p*‐value 0.027). The relationship was stronger with logarithmic analysis with a 1‐unit increase in the log of the SUVmax corresponding with a doubling (odds ratio 2.038) of the odds of having distant metastases (*p*‐value 0.001) (Table 2 and Figure 4).

It was noted, however, that distant metastasis was noted in patients with a primary SUVmax of as low as 5.3 while a patient with a primary SUVmax of 117 did not demonstrate distant metastases (Table 1 and Figure 2).

### 3.4 Nodal or Distant Metastasis

When the presence of nodal or distant metastases was analysed together, 85 patients (approximately 32% of the cohort) fell into this category. The median SUVmax of the primary prostate cancer of those patients without metastatic disease was 12.0 compared with 18.7 for patients with PSMA‐avid nodal metastases. The mean SUVmax was 17.3 versus 23.8, respectively. Both of these findings were clinically significant with a *p*‐value of < 0.001 (Table 1).

**FIGURE 5:**
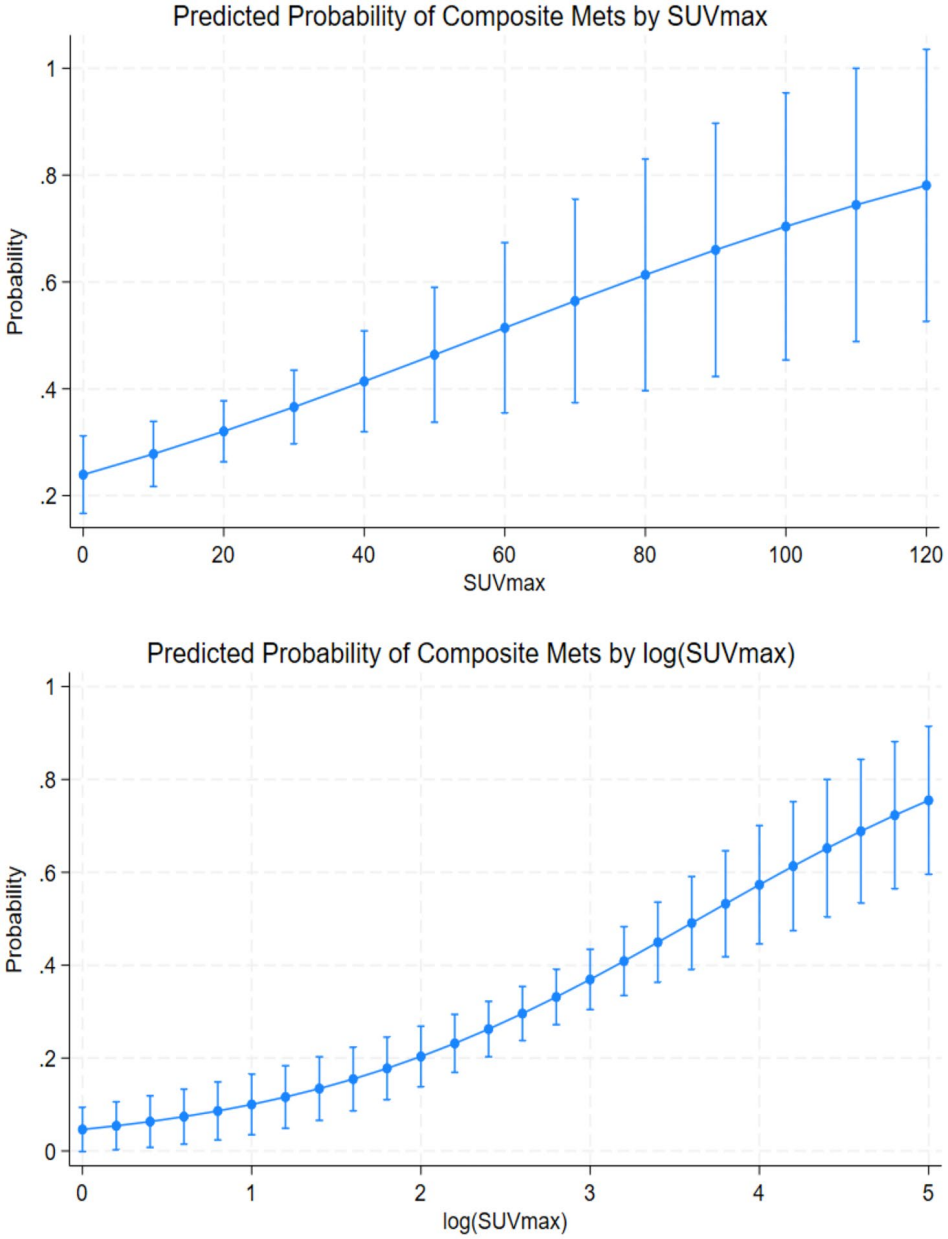
Linear and logarithmic analysis of SUVmax correlated with the probability of any metastasis (with 95% confidence intervals).

Linear regression modelling demonstrates that a 1‐unit increase in SUVmax corresponds with a 2% increase in the odds of having any metastatic disease. While the logarithmic regression modelling demonstrates a 1‐unit increase in the log of the SUVmax corresponds with a greater than doubling (odds ratio 2.294) of the odds of having any metastatic disease (*p*‐value < 0.001) (Table 2 and Figure 5).

## 4 Discussion

With the 265 patient cohort who underwent primary staging of biopsy‐proven prostate cancer with 68Ga‐PSMA PET/CT, we have been able to establish a relationship between SUVmax of the primary lesion and the presence of nodal or distant metastasis. Although statistically significant, this relationship is very weak, with each unit increase in SUVmax only corresponding to a less than 2% increase in odds of metastatic disease. This relationship is stronger when correlated with the log of the SUVmax in all subgroup analyses, with a doubling of the odds with each unit of increase. But the log of the SUVmax is of limited clinical use as, in a practical setting, the SUVmax of the primary prostate lesion is rarely greater than 100 (which would result in log(SUVmax) of 2).

The significant overlap in SUVmax values between localised and advanced disease is not unexpected as previous studies have shown that PSMA expression in the primary tumour does not correlate with disease mortality in surgically treated patients [4]. Furthermore, the expression of PSMA may reduce with more aggressive cancers due to the dedifferentiation of the primary lesion resulting in reduced PSMA expression and uptake [5]. As such, patients who have biopsy‐proven prostate cancer, but minimal PSMA uptake in the primary lesion may also require further imaging (such as 18F FDG PET) to more accurately assess the presence of distant disease [6].

Given that SUVmax is related to both the size of the lesions and receptor expression [7], the presence of micrometastatic disease that is below the threshold for detection may also result in the underestimation of patients with metastatic disease. However, this would presumably strengthen the relationship between the SUVmax and metastasis status, as metastatic disease in those with only mildly avid, PSMA‐avid primaries is more likely to be missed on 68Ga‐PSMA PET/CT. This would require further study with correlation to histological samples in patients who have undergone radical prostatectomy and bilateral iliac lymph node dissections and is outside the scope of this study.

An ideal outcome would have been to see a sharp increase in the rate of nodal and distant metastases at a specific cut‐off SUVmax value, which would then allow empiric treatment for presumed metastatic disease. The weak relationship between the SUVmax and advanced disease suggests that a hard cut‐off value will both miss metastatic disease in some, while leading to overtreatment in others.

As this was a retrospective study, the patients who were included in this dataset had already undergone management (based on initial imaging results) at the time of data collection. Further analysis into the histological samples from patients who underwent radical prostatectomy and lymph node dissection was not performed in this study but may be beneficial in assessing radiological/pathological correlation.

Although the SUVmax of the primary lesion does not correlate strongly with the presence of distant metastases, it remains a useful tool in describing the strength of PSMA expression of prostate cancer. This becomes relevant with the advent of theranostics with the increasing use of Lutetium‐177‐PSMA for treatment of castrate resistant prostate cancer [8].

## 5 Conclusion

There is a weak but statistically significant correlation between the SUVmax of the primary prostate lesion and the presence of metastases on primary staging 68Ga‐PSMA PET/CT for biopsy‐proven prostate cancer. However, because the relationship is so weak and the significant overlap in SUVmax in local versus advanced disease, using SUVmax as the sole predictor of advanced disease is unreliable.

## Author Contributions

**Geoffrey Liu:** conceptualization, methodology, software, data curation, investigation, validation, formal analysis, visualization, project administration, resources, writing – original draft, writing – review and editing.

## Conflicts of Interest

The authors declare no conflicts of interest.

## Data Availability

The data that support the findings of this study are available on request from the corresponding author. The data are not publicly available due to privacy or ethical restrictions.
